# Relationship between the hemoglobin-to-red cell distribution width ratio and post-stroke cognitive impairment: a prospective study

**DOI:** 10.3389/fnagi.2025.1552956

**Published:** 2025-04-30

**Authors:** Yufeng Liu, Zhe Xie, Ping Wang, Fan Liu, Liandong Zhao, Chun Chen, Qiu Ge, Mengchao Wang, Zhongwen Zhi

**Affiliations:** Department of Neurology, The Affiliated Huai'an Hospital of Xuzhou Medical University and The Second People's Hospital of Huai'an, Huai'an, China

**Keywords:** stroke, cognitive impairment, hemoglobin, red cell distribution width, HRR

## Abstract

**Background:**

Post-stroke cognitive impairment (PSCI) is one of the main complications after stroke. The association between the hemoglobin-to-red cell distribution width ratio (HRR) and PSCI remains inadequately explored. Consequently, we performed a prospective study to assess whether HRR levels are associated with changes in cognitive function after acute ischemic stroke (AIS).

**Methods:**

A total of 296 AIS patients were recruited. HRR was measured within 24 h of admission, and cognitive function was assessed using the Mini-Mental State Examination (MMSE) one month post-onset. Logistic regression analysis was performed to identify independent risk and protective factors for the occurrence of PSCI. Restricted cubic splines (RCS) were used to explore the dose-response relationship between HRR and PSCI.

**Results:**

129 of 296 participants (43.6%) developed cognitive impairment at 1 month. HRR in PSCI group was significantly lower than that in non-cognitive impairment group (*P* < 0.001). When HRR was taken as the categorical variable and with Q4 as the reference, the risk of PSCI in Q1 was the highest after adjusting multiple potential confounding factors (odds ratio [OR] = 2.702, 95% confidence interval [CI]= 1.222–5.977, *P* = 0.014). In addition, RCS curve exhibited that the relationship between HRR and PSCI was linear (*P* for nonlinear = 0.972, *P* for overall = 0.012). Subgroup analysis verified the stability of the results.

**Conclusion:**

Reduced HRR levels were linked to an increased risk of cognitive impairment, indicating that HRR may serve as a predictive factor for PSCI.

## 1 Introduction

Stroke continues to be the primary cause of disability (Ma et al., 2021). Post-stroke cognitive impairment (PSCI) is a prevalent post-stroke complication, impacting between 25% and 81% of patients (Sun et al., 2014). Previous studies have indicated that the presence of cognitive impairment is associated with poorer functional outcomes and a higher risk of recurrent stroke (Lee et al., 2014; Synhaeve et al., 2015), and that PSCI significantly affects patients' neurological recovery and survival time (Rost et al., 2022). Furthermore, stroke survivors will face heightened medical expenses due to the aftermath. Consequently, new predictive biomarkers are being explored to improve the identification and early diagnosis of PSCI, thereby potentially offering fresh perspectives on treatment strategies for PSCI.

Red blood cells are the most important medium for transporting oxygen in blood and are closely related to the occurrence and development of ischemic stroke (IS). Both anemia and low hemoglobin levels are believed to elevate the risk of stroke. Additionally, abnormal hemoglobin levels (anemia and high hemoglobin) are linked to increased risks of ischemic cerebrovascular disease, including all-cause mortality, poor functional outcomes, stroke recurrence, and complex vascular events (Zhang et al., 2021).

Red cell distribution width (RDW) is an important parameter that indicates variability in red blood cell volume and is accessible through routine blood tests. Clinically, RDW is commonly used in combination with other hematological indicators to diagnose blood disorders. Recent studies have shown that variations in RDW levels are also associated with the neuroendocrine system, chronic inflammation, oxidative stress, and other factors. RDW is associated with various diseases and serves as an independent and strong risk factor for mortality from these conditions (Wang et al., 2020; Ding et al., 2022; Liang et al., 2022). A cohort study of healthy individuals found that RDW levels showed a positive correlation with IS incidence and common carotid intima-media thickness. This relationship may be attributed to systemic inflammation, nutritional deficiencies, and increased renin-angiotensin system activation (Söderholm et al., 2015). Studies have shown that increased RDW can predict stroke severity and unfavorable functional outcomes in patients with IS (Kara et al., 2015; Xue et al., 2022). Similarly, elevated RDW levels were linked to a higher mortality risk following acute ischemic stroke (AIS) (Shen and Shen, 2024).

The hemoglobin-to-red cell distribution width ratio (HRR) serves as a novel biomarker for inflammation, calculated using hemoglobin and RDW. HRR levels exhibit a negative association with stroke incidence; each unit increase in HRR decreases the likelihood of a stroke by 58% (Xiong et al., 2024). The lower the level of HRR, the worse the functional prognosis at 3 months and the higher the mortality in IS patients, and this relationship is negative nonlinear (Qin et al., 2022; Xie et al., 2024). RDW may be associated with cognitive function and has been found to be elevated in individuals with Alzheimer's disease (AD) and vascular dementia (VaD) (Öztürk et al., 2013; Jiang et al., 2021). Likewise, the association between anemia and cognitive impairment is well-documented (Winchester et al., 2018). High level of hemoglobin at baseline may decrease the incidence of PSCI and serve as a protective factor for cognitive function (Jia et al., 2022).

However, studies exploring the relationship between HRR and PSCI remain insufficient. We hypothesize that HRR might influence cognitive dysfunction risk in AIS patients. AIS patients were recruited to have their HRR levels measured on admission and then cognitive assessment was performed one month after stroke to investigate the association between HRR and PSCI.

## 2 Materials and methods

### 2.1 Study design and population

A prospective cohort study was conducted in which patients with AIS were admitted to the Department of Neurology of the affiliated Huai'an Hospital of Xuzhou Medical University between January and October 2023. Patients who met the criteria were included, and cognitive assessment was performed using the Mini-Mental State Examination (MMSE) 1 month after stroke. Venous blood samples were taken within 24 h of admission to assess HRR and other indicators. Clinical data was also recorded during hospitalization for analysis.

The inclusion criteria were as follows: (1) Patients met the diagnostic criteria of AIS and were confirmed by computed tomography or magnetic resonance imaging at admission; (2) Time from onset to admission ≤ 7 days; (3) Age between 18 and 80 years old; (4) Have the ability and willingness to complete regular follow-up, voluntarily signed informed consent.

The exclusion criteria were as follows: (1) Combined with other serious central nervous system diseases, such as Parkinson's disease or hydrocephalus; (2) Significant cognitive dysfunction before stroke; (3) Patients with severe speech, hearing, and visual impairment, intolerance, or cooperation with relevant examinations of this study; (4) Patients with a history of depression, anxiety, schizophrenia or long-term use of psychotropic drugs; (5) Severe liver, kidney, lung dysfunction, acute and chronic inflammatory diseases and other acute vascular ischemic diseases including acute myocardial infarction; (6) Previous autoimmune diseases, malignant tumors, and hematological diseases.

### 2.2 Data collection

Demographic characteristics, vascular risk factors, clinical data, and laboratory data were collected after admission. Including gender, age, education, body mass index (BMI), hypertension, diabetes, coronary heart disease, atrial fibrillation, previous stroke, smoking, drinking, and anemia (defined as hemoglobin <130 g/L for males and <120 g/L for females). The National Institutes of Health Stroke Scale (NIHSS) was used to assess the degree of neurological deficits at admission. The causes of stroke were collected (TOAST mechanism), and white blood cell count (WBC), hemoglobin, RDW, platelet count, mean corpuscular volume (MCV), and glycated hemoglobin (HbA1c) were recorded. HbA1c levels were reported in the National Glycohemoglobin Standardization Program (NGSP) units (A1c). According to the formula developed by Hoelzel et al. (2004), we convert the A1c value to the International Federation of Clinical Chemistry and Laboratory Medicine (IFCC) units (iA1c). Finally, iA1c was put into the formula developed by Koga et al. (2023) to calculate the estimated mean erythrocyte age (Mrbc) (which is approximately half of the red blood cells lifespan). The calculation formula involved is as follows: HRR = hemoglobin (g/L)/RDW (%); iA1c (mmol/mol) = 10.93 × HbA1c_NGSP (%) – 23.53; Mrbc (days) = 1.45 × iA1c (mmol/mol).

### 2.3 Evaluation of cognitive function

The patients were followed up at 1 month after stroke, two professionally trained neurologists used the MMSE Beijing version to evaluate the patients in a quiet environment. MMSE score range from 0 to 30, with higher scores indicating better cognitive function. According to the MMSE score, participants were divided into PSCI group and post-stroke non-cognitive impairment (PSNCI) group. In this study, patients with an MMSE score ≤ 17 points (illiterate), ≤ 20 points (years of education ≤ 6) or ≤ 24 points (years of education > 6) were considered to have cognitive impairment (Liu et al., 2017). The cognitive assessment was conducted by two trained physicians and consistency testing was performed prior to the start of the study to ensure the reliability of the assessment (Simple KAPPA Coefficient: 0.898).

### 2.4 Statistical analysis

Participants were divided into quartiles according to their HRR levels upon admission: Quartile1 (≤9.70), Quartile2 (9.71–10.73), Quartile3 (10.74–11.71), and Quartile4 (≥11.72). Continuous variables were expressed as means with standard deviation (SD) or median with interquartile range (IQR). Categorical variables were described using frequencies and percentages. Differences in baseline characteristics in the patients with and without were compared using an independent sample *t*-test, Pearson Chi-square test, Fisher's exact test, one-way analysis of variance (Bonferroni *post-hoc* test), and a Kruskal–Wallis H-test. Binary logistic regression analysis was used to detect the relationship between HRR and PSCI, and the adjusted covariates were variables with a *P* < 0.05 in univariate analysis. We established three logistic regression models, model 1: unadjusted; model 2: adjusted for gender, age, and education; model 3: adjusted for covariates from model 2 and further adjusted for atrial fibrillation, previous stroke, NIHSS, white blood cell count, and platelet count. Spline regression models were used to explore the shape of the association between HRR and PSCI, fitting a restricted cubic spline function with 3 knots (at the 5th, 50th, and 95th percentiles). In addition, patients were divided into subgroups based on gender, age (<65; ≥65), hypertension, diabetes, coronary heart disease, atrial fibrillation, smoking, drinking, anemia, and previous stroke. Subgroup analysis was used to verify the stability of the results. Correlation analyses were performed using Pearson correlation or Spearman rank-order correlation.

All the research data were analyzed by the SPSS (version 25.0), and R software (version 4.3.2). Two-tailed *P* < 0.05 was considered statistically significant.

## 3 Results

### 3.1 Study recruitment profile

**Figure 1:**
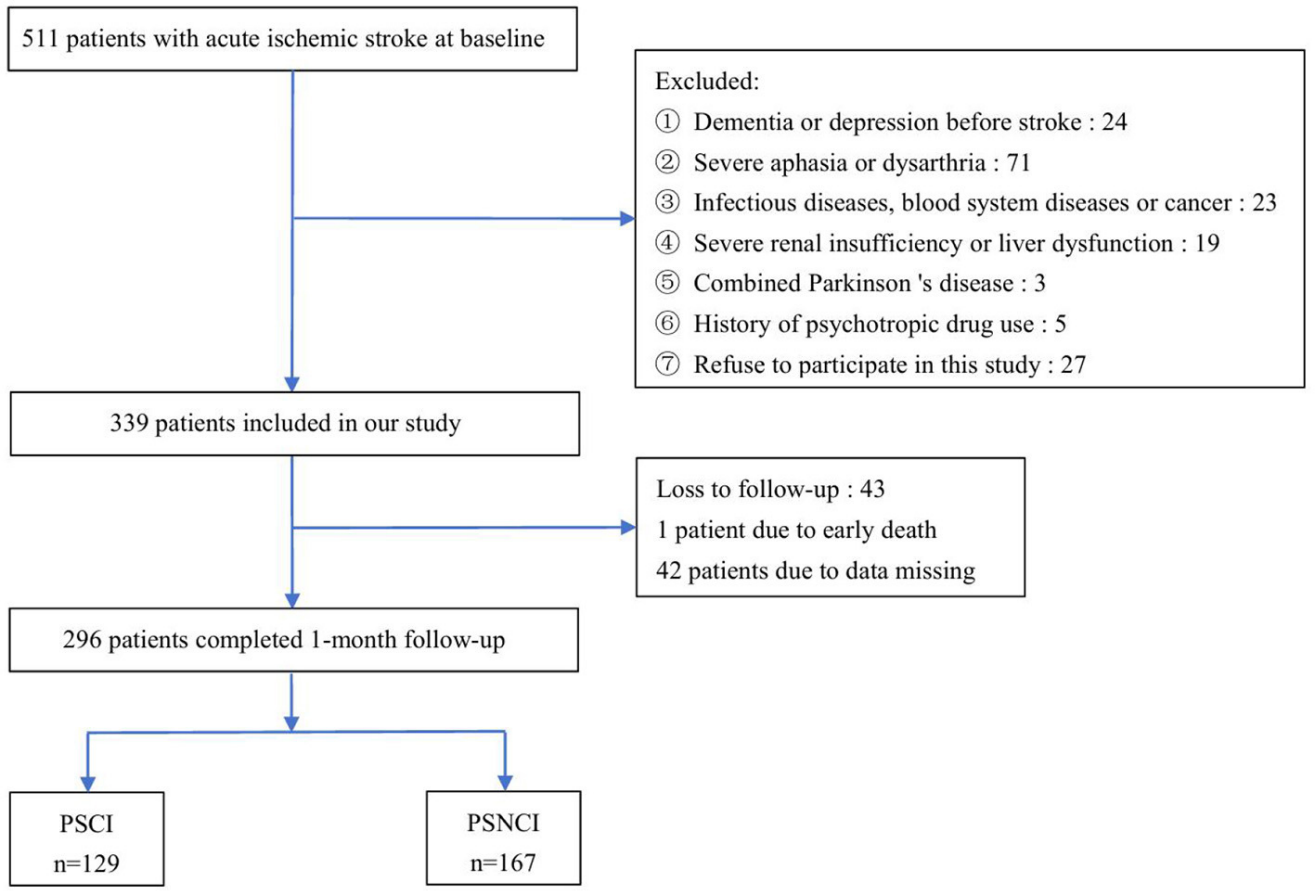
Study recruitment profile. PSCI, post-stroke cognitive impairment; PSNCI, post-stroke non-cognitive impairment.

A total of 511 AIS patients were enrolled at baseline and 339 met the inclusion criteria. During the 1-month follow-up, 1 patient died, and 42 patients were lost to follow-up due to missing data. Finally, 296 patients completed the follow-up and were included in the statistical analysis, and 129 patients were considered to have cognitive impairment. The incidence of PSCI in this study was 43.6% (Figure 1).

### 3.2 Baseline characteristics of patients with PSCI and PSNCI

Table 1 shows the comparison of general baseline data between the PSCI group and the PSNCI group, and the HRR of patients in the PSCI group was significantly lower than that in the PSNCI group (*P* < 0.001). Additionally, gender, age, education, atrial fibrillation, previous stroke, NIHSS, white blood cell count, and platelet count showed statistically significant differences (all *P* < 0.05) (Table 1). To further evaluate the relationship between HRR stratification and PSCI, patients were categorized into four subgroups according to HRR levels: Q1(≤9.70), Q2 (9.71-10.73), Q3 (10.74-11.71), and Q4 (≥11.72). Significant differences in PSCI incidence were observed among the four groups (*P* < 0.001). Furthermore, gender, age, BMI, drinking, previous stroke, anemia, TOAST mechanism, NIHSS, hemoglobin, RDW, and MMSE score differed significantly across all groups (all *P* < 0.05) (Table 2).

**Table 1:** Baseline characteristics of patients with PSCI and PSNCI.

**Variables**	**All patients**	**PSNCI patients**	**PSCI patients**	***P*-value**
	**(*n* = 296)**	**(*n* = 167)**	**(*n* = 129)**	
Female, *n* (%)	113 (38.2)	53 (31.7)	60 (46.5)	0.009
Age (years), mean ± SD	66.46 ± 10.04	65.20 ± 10.26	68.10 ± 9.54	0.013
Education, *n* (%)				0.005
Illiterate	114 (38.5)	54 (32.3)	60 (46.5)	
Years of education ≤ 6	92 (31.1)	50 (29.9)	42 (32.6)	
Years of education > 6	90 (30.4)	63 (37.7)	27 (20.9)	
BMI (kg/m^2^), mean ± SD	24.85 ± 3.77	25.07 ± 3.90	24.56 ± 3.58	0.247
Hypertension, *n* (%)	223 (75.3)	124 (74.3)	99 (76.7)	0.622
Diabetes, *n* (%)	99 (33.4)	51 (30.5)	48 (37.2)	0.228
Coronary heart disease, *n* (%)	63 (21.3)	39 (23.4)	24 (18.6)	0.322
Atrial fibrillation, *n* (%)	41 (13.9)	17 (10.2)	24 (18.6)	0.037
Smoking, *n* (%)	72 (24.3)	35 (21.0)	37 (28.7)	0.125
Drinking, *n* (%)	59 (19.9)	31 (18.6)	28 (21.7)	0.502
Previous stroke, *n* (%)	46 (15.5)	18 (10.8)	28 (21.7)	0.010
Anemia, *n* (%)	67 (22.6)	32 (19.2)	35 (27.1)	0.104
TOAST mechanism, *n* (%)				0.187
LAA	185 (62.5)	105 (62.9)	80 (62.0)	
CE	37 (12.5)	16 (9.6)	21 (16.3)	
SAA	67 (22.6)	43 (25.7)	24 (18.6)	
ESUS	7 (2.4)	3 (1.8)	4 (3.1)	
NIHSS (score), median (IQR)	2 (1–5)	2 (1–4)	3 (1–7)	0.029
WBC (×10^9^/L), median (IQR)	7.23 (5.69–9.28)	6.90 (5.66–8.73)	7.59 (6.01–9.61)	0.045
Platelet count (×10^9^/L), median (IQR)	190 (162–228)	185 (156–225)	197 (166–245.5)	0.036
Hemoglobin (g/L), mean ± SD	137.58 ± 19.52	140.91 ± 18.99	133.27 ± 19.43	<0.001
RDW (%), mean ± SD	13.15 ± 1.40	12.89 ± 1.04	13.48 ± 1.72	<0.001
HRR, mean ± SD	10.57 ± 1.82	11.00 ± 1.72	10.02 ± 1.80	<0.001
MCV (fL), mean ± SD	89.86 ± 5.88	90.27 ± 6.02	89.32 ± 5.67	0.170
HbA1c (%), median (IQR)	6.00 (5.40–6.88)	5.90 (5.40–6.70)	6.00 (5.45–7.05)	0.577
iA1c (mmol/mol), median (IQR)	42.05 (35.49–51.61)	40.96 (35.49–49.70)	42.05 (36.04–53.63)	0.577
Mrbc (days), median (IQR)	60.97 (51.46–74.84)	59.39 (51.46–72.07)	60.97 (52.26–77.61)	0.577

SD, Standard deviation; IQR, Interquartile range; PSCI, Post-stroke cognitive impairment; PSNCI, Post-stroke non-cognitive impairment; BMI, Body mass index; LAA, Large artery atherosclerosis; CE, Cardioembolism; SAO, Small-artery occlusion; ESUS, Embolic stroke of undetermined source; NIHSS, National Institutes of Health Stroke Scale; WBC, White blood cell count; RDW, Red cell distribution width; HRR, Hemoglobin-to-red cell distribution width ratio; MCV, Mean corpuscular volume; A1C: HbA1c expressed in National Glycohemoglobin Standardization Program (NGSP) units; iA1c: HbA1c expressed in International Federation of Clinical Chemistry and Laboratory Medicine (IFCC) units; Mrbc, mean erythrocyte age.

**Table 2:** Baseline characteristics of all patients in HRR quartiles.

**Variables**	**HRR quartiles**				***P*-value**
	≤**9.70**	**9.71-10.73**	**10.74-11.71**	≥**11.72**	
	***n*** = **74**	***n*** = **74**	***n*** = **74**	***n*** = **74**	
Female, *n* (%)	38 (51.4)	39 (52.7)	22 (29.7)	14 (18.9)	<0.001
Age (years), median (IQR)	72.50 (65.00–76.00)	72.50 (61.75–76.00)	69.00 (60.00–73.00)	62.00 (53.00–69.00)	<0.001
Education, *n* (%)					0.149
Illiterate	27 (36.5)	32 (43.2)	33 (44.6)	22 (29.7)	
Years of education ≤ 6	27 (36.5)	24 (32.4)	21 (28.4)	20 (27)	
Years of education > 6	20 (27.0)	18 (24.3)	20 (27)	32 (43.2)	
BMI (kg/m^2^), median (IQR)	23.13 (21.02–26.09)	25.14 (22.69–27.34)	24.82 (23.21–27.28)	25.64 (23.09–27.68)	0.002
Hypertension, *n* (%)	55 (74.3)	56 (75.7)	57 (77.0)	55 (74.3)	0.978
Diabetes, *n* (%)	26 (35.1)	24 (32.4)	24 (32.4)	25 (33.8)	0.983
Coronary heart disease, *n* (%)	10 (13.5)	16 (21.6)	15 (20.3)	22 (29.7)	0.118
Atrial fibrillation, *n* (%)	15 (20.3)	12 (16.2)	5 (6.8)	9 (12.2)	0.102
Smoking, *n* (%)	17 (23.0)	15 (20.3)	16 (21.6)	24 (32.4)	0.299
Drinking, *n* (%)	7 (9.5)	10 (13.5)	17 (23.0)	25 (33.8)	0.001
Previous stroke, *n* (%)	20 (27.0)	10 (13.5)	11 (14.9)	5 (6.8)	0.007
Anemia, *n* (%)	54 (73.0)	13 (17.6)	0 (0)	0 (0)	<0.001
TOAST mechanism, *n* (%)					0.043
LAA	51 (68.9)	46 (62.2)	49 (66.2)	39 (52.7)	
CE	14 (18.9)	10 (13.5)	5 (6.8)	8 (10.8)	
SAA	9 (12.2)	16 (21.6)	18 (24.3)	24 (32.4)	
ESUS	0 (0)	2 (2.7)	2 (2.7)	3 (4.1)	
NIHSS (score), median (IQR)	3.50 (2.00–6.25)	2.00 (1.00–6.25)	2.00 (1.00–4.00)	2.00 (1.00–4.00)	0.011
WBC (×10^9^/L), median (IQR)	7.30 (5.69–9.30)	7.01 (5.89–9.48)	6.99 (5.60–9.17)	7.31 (5.69–9.27)	0.971
Platelet count (×10^9^/L), median (IQR)	188.50 (153.50–228.00)	199.50 (167.00–244.25)	182.00 (159.25–236.00)	190.00 (169.75–224.25)	0.536
Hemoglobin (g/L), mean ± SD	114.14 ± 16.11	133.64 ± 9.97	144.53 ± 6.07	158.03 ± 9.94	<0.001
RDW (%), mean ± SD	14.09 ± 2.23	13.14 ± 0.91	12.83 ± 0.53	12.53 ± 0.69	<0.001
MCV (fL), mean ± SD	88.85 ± 6.67	90.63 ± 6.75	89.52 ± 4.73	90.43 ± 5.01	0.224
HbA1c (%), median (IQR)	5.80 (5.25–6.38)	6.10 (5.68–6.85)	5.80 (5.48–7.13)	6.10 (5.30–7.43)	0.079
iA1c (mmol/mol), median (IQR)	39.86 (33.85–46.15)	43.14 (38.50–51.34)	39.86 (36.31–54.35)	43.14 (34.40–57.63)	0.079
Mrbc (days), median (IQR)	57.80 (49.09–66.92)	62.56 (55.82–74.44)	57.80 (52.65–78.80)	62.56 (49.88–83.56)	0.079
PSCI, *n* (%)	46 (62.2)	36 (48.6)	27 (36.5)	20 (27.0)	<0.001
MMSE (score), median (IQR)	19 (14–24)	19 (14–26)	23 (16–27)	26 (19–29)	<0.001

SD, Standard deviation; IQR, Interquartile range; HRR, Hemoglobin-to-red cell distribution width ratio; BMI, Body mass index; LAA, Large artery atherosclerosis; CE, Cardioembolism; SAO, Small-artery occlusion; ESUS, Embolic stroke of undetermined source; NIHSS, National Institutes of Health Stroke Scale; WBC, White blood cell count; RDW, Red cell distribution width; MCV, Mean corpuscular volume; A1C: HbA1c expressed in National Glycohemoglobin Standardization Program (NGSP) units; iA1c: HbA1c expressed in International Federation of Clinical Chemistry and Laboratory Medicine (IFCC) units; Mrbc, mean erythrocyte age; PSCI, Post-stroke cognitive impairment; MMSE, Mini mental state examination.

### 3.3 Association between HRR and PSCI

**Figure 2:**
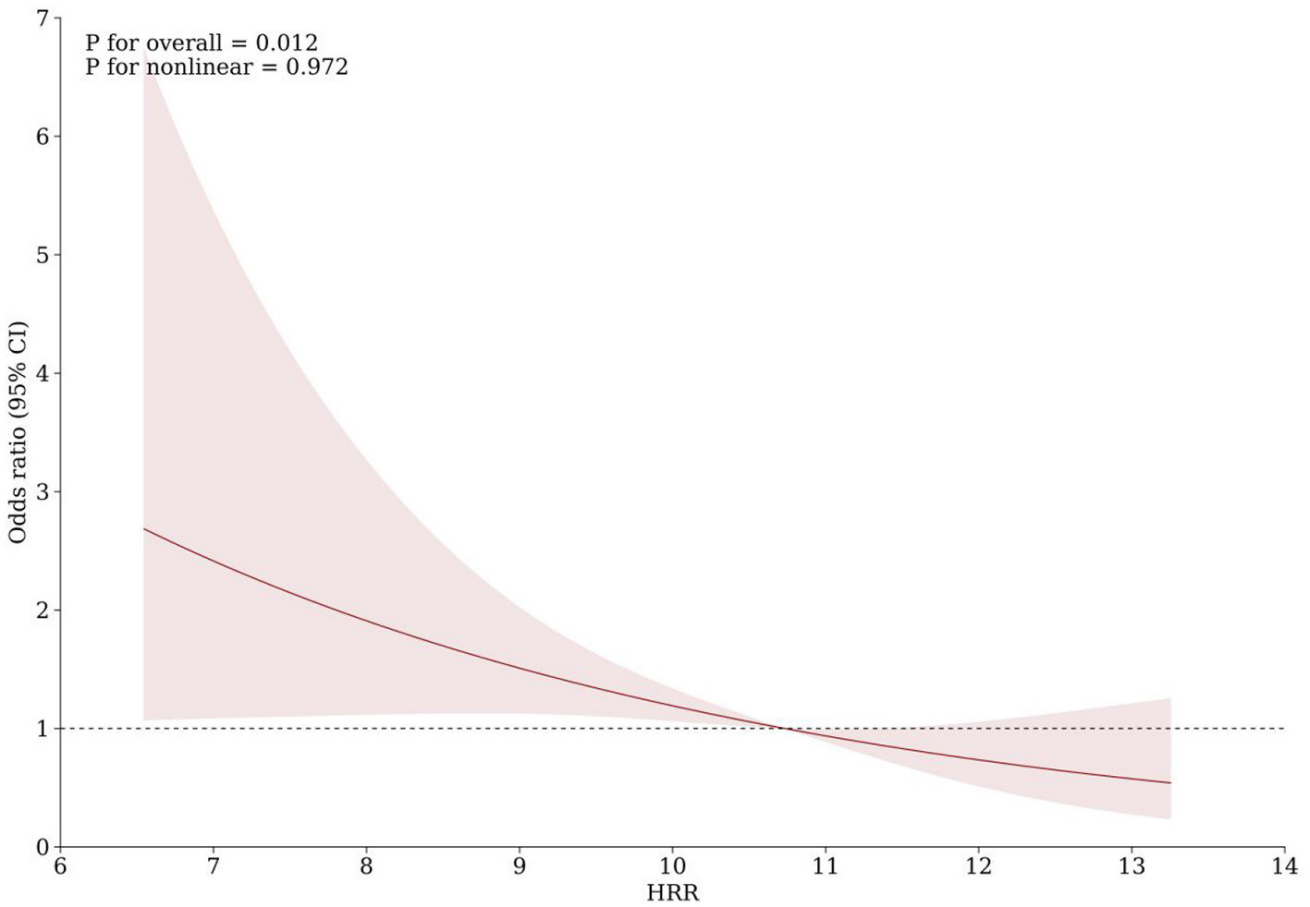
Association of HRR with risk of cognitive impairment after acute ischemic stroke. Odds ratios and 95% confidence intervals derived from restricted cubic spline regression, with knots placed at the 5th, 50th, and 95th percentiles of the distribution of HRR. The reference point is the median of HRR (10.73). Odds ratios were adjusted for the same covariates as model 3 in Table 3.

**Figure 3:**
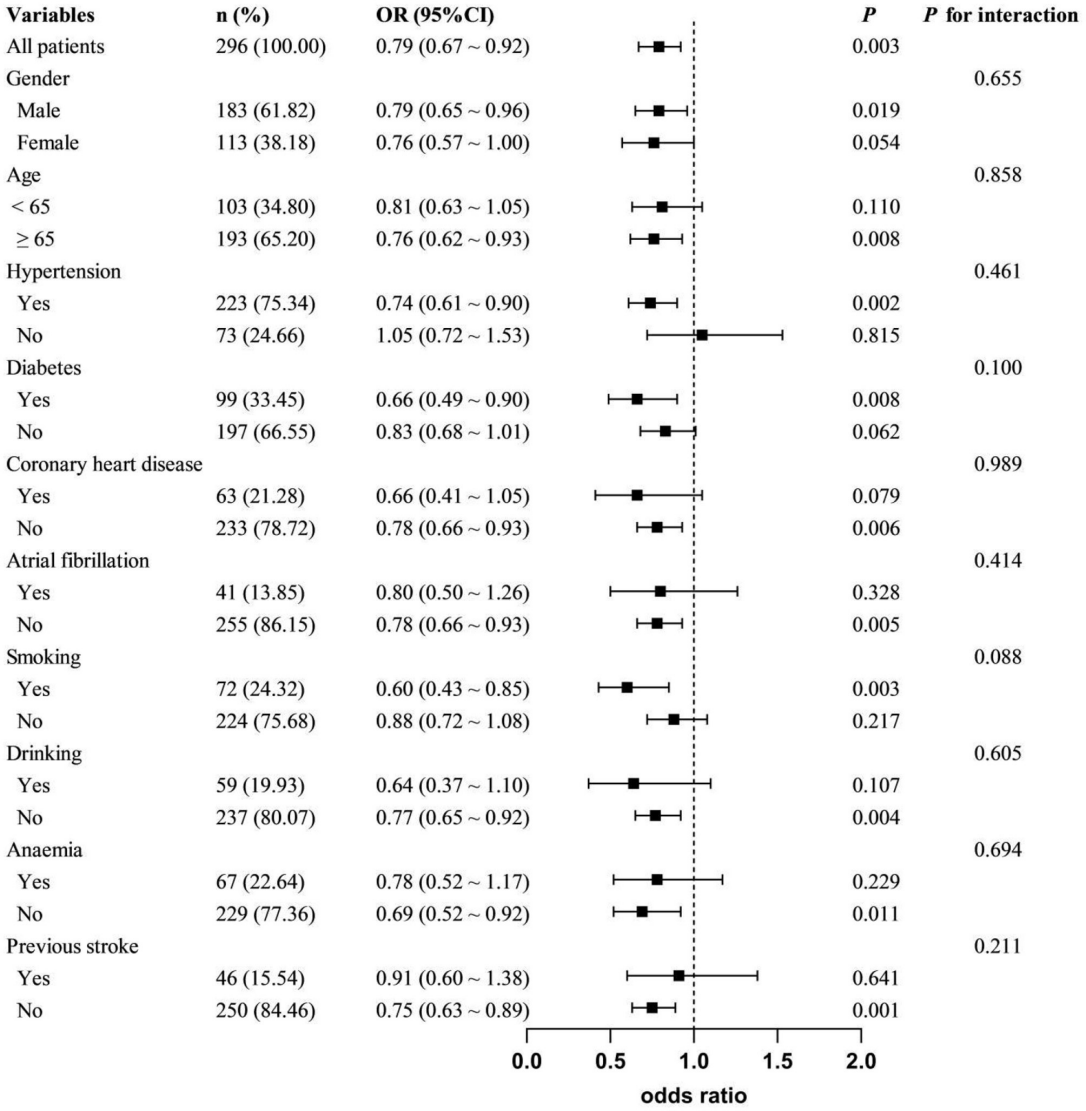
Subgroup analyses of the association between HRR and PSCI. Adjusted covariates included gender, age, education, atrial fibrillation, previous stroke, NIHSS, white blood cell count, and platelet count, except for the stratified variable.

As shown in Table 3, with the highest quartile of HRR as reference, patients in the first quartile of HRR had a significantly increased risk of PSCI (Model 1: OR = 4.436, 95%CI = 2.212-8.894, *P* < 0.001; Model 2: OR = 3.189, 95%CI = 1.495-6.804, *P* = 0.003; Model 3: OR =2.702, 95%CI = 1.222–5.977, *P* = 0.014). When used as a continuous variable, HRR level at admission was still correlated with PSCI, and confounding factors were controlled (Model 1: OR = 0.722, 95%CI = 0.625–0.834, *P* < 0.001; Model 2: OR = 0.760, 95%CI = 0.654–0.884, *P* < 0.001; Model 3: OR = 0.788, 95%CI = 0.673–0.921, *P* = 0.003). The trend analysis indicated that HRR was negatively correlated with the occurrence of PSCI (Table 3). In addition, the RCS curve revealed a linear relationship between HRR and PSCI with the adjustment of Model 3 (*P* for nonlinear = 0.972, *P* for overall = 0.012) (Figure 2). Further subgroup analysis showed no significant interactions between HRR levels and potential effect modifiers after adjusting for covariates in model 3 (all *P* for interaction > 0.05) (Figure 3).

**Table 3:** Multivariate logistic analysis for the association between HRR levels and PSCI.

**HRR**	**PSCI**	**Model 1**		**Model 2**		**Model 3**	
	***n*** **(%)**	**OR (95%CI)**	***P*** **-value**	**OR (95%CI)**	***P*** **-value**	**OR (95%CI)**	***P*** **-value**
Quartile1 (8.60[7.40–9.32])	46 (62.2)	4.436 (2.212–8.894)	<0.001	3.189 (1.495–6.804)	0.003	2.702 (1.222–5.977)	0.014
Quartile2 (10.19[9.92–10.40])	36 (48.6)	2.558 (1.288–5.081)	0.007	1.765 (0.838–3.721)	0.135	1.489 (0.685–3.237)	0.315
Quartile3 (11.29[11.04–11.53])	27 (36.5)	1.551 (0.772–3.117)	0.218	1.199 (0.577–2.492)	0.626	1.174 (0.552–2.499)	0.677
Quartile4 (12.40[12.13–12.84])	20 (27.0)	**Reference**		**Reference**		**Reference**	
*P* for trend			<0.001		0.001		0.008

OR, Odds ratio; CI, Confidence interval; HRR, Hemoglobin-to-red cell distribution width ratio; PSCI, Post-stroke cognitive impairment; Reference OR (1.000) is the highest Quartile of HRR for PSCI; Model 1: unadjusted; Model 2: adjusted for gender, age, and education; Model 3: adjusted for covariates from model 2 and further adjusted for atrial fibrillation, previous stroke, NIHSS, white blood cell count, and platelet count.

## 4 Discussion

The primary finding of this study is that low HRR is an independent risk factor for cognitive impairment one month after stroke. As HRR levels increase, the risk of PSCI decreases, suggesting a protective role of HRR against cognitive impairment. The RSC curve indicates a linear relationship between HRR and PSCI, and additional subgroup analyses confirm the stability of these results. Thus, this study demonstrates that HRR is a novel and independent predictor of PSCI risk stratification.

Anemia is recognized as a significant, modifiable risk factor contributing to cognitive decline. Reduced hemoglobin levels were correlated with cognitive decline and VaD in elderly individuals (Andro et al., 2013; Tan et al., 2018). Jia et al. (2022) found that baseline hemoglobin levels were an independent protective factor for PSCI. However, the association between hemoglobin and PSCI within specific subgroups, such as gender, remains uncertain due to the absence of subgroup analysis. Based on the moderate negative correlation between RDW and MMSE score, RDW was considered to be positively correlated with the degree of cognitive impairment in AD patients (Öztürk et al., 2013). The MIND-China Study (Jiang et al., 2021) identified a similar J-shaped relationship between RDW and the odds ratios for AD and VaD, particularly in individuals without anemia. Weuve et al. (2014) found that for each 1 unit (%) increase in RDW, there is a 6% increase in the risk of dementia among the elderly. While anemia, RDW, and cognitive impairment have been extensively explored, HHR and PSCI remain under-studied. Our findings indicate that patients with reduced HHR levels have an increased risk of cognitive impairment. Moreover, the trend analysis and the RCS model reveal a negative linear relationship between the two. Given the physiological differences, female patients were prone to lower hemoglobin levels and anemia. Therefore, we conducted a gender-based subgroup analysis and discovered a stronger association in male (OR = 0.79; 95% CI: 0.65–0.96), albeit the difference was not statistically significant (*P* for interaction = 0.655). Previous studies have shown that RDW levels appear to be more susceptible to anemia, and thus manifest differences in cognitive impairment outcomes. However, our study did not detect a statistically significant interaction between anemic and non-anemic groups (*P* for interaction = 0.694). A previous stroke significantly influences the incidence of PSCI, with a notably higher proportion of stroke history observed in the PSCI group. We found significant differences in stroke history among groups with varying HRR levels (*P* = 0.007). Therefore, it is necessary to clarify whether stroke history affects the relationship between HRR and PSCI. Subgroup analysis showed a stronger association between HRR and PSCI in first-time stroke patients (OR = 0.75, 95% CI: 0.63–0.89, *P* = 0.001). However, no significant interaction was detected (*P* for interaction = 0.211). This suggests that HRR may be more broadly applicable for predicting PSCI in various populations. The sample size in this study is relatively small, especially in terms of the number of individuals with anemia. Therefore, further studies with larger sample sizes are needed to confirm our findings.

HRR may be involved in the occurrence and development of PSCI through diverse mechanisms. Decreased hemoglobin levels were associated with structural changes in the brain. Anemia and severe white matter impairment have been found to be associated with executive dysfunction in patients with mild cognitive impairment, suggesting that anemia is involved in the development of white matter lesions (Son et al., 2012). Low hemoglobin levels were also linked to lobular microbleeds and atrophy of the occipital cortex (Tan et al., 2018). Low hemoglobin and lack of hematopoietic raw materials can result in nervous system degeneration, altered iron metabolism, and elevated oxidative stress within brain tissue (Dröge and Schipper, 2007). Anemia induces chronic cerebral hypoxia, potentially contributing to cognitive decline by accelerating the accumulation of Abeta, enhancing tau hyperphosphorylation, disrupting the blood-brain barrier, and facilitating neuronal degeneration (Zhang and Le, 2010). Additionally, anemia can be accompanied by a deficiency of hematopoietic raw materials, inflammation, and malnutrition. These factors have also been validated in studies of PSCI (Lee et al., 2021; Shang et al., 2022; Xu et al., 2023). RDW represents the uniformity of the red blood cell volume size in the blood. An elevated RDW level suggests disruption in red blood cell homeostasis, potentially due to various metabolic abnormalities including malnutrition, cellular inflammation, telomere alterations, and oxidative stress (Salvagno et al., 2015). A series of studies have established RDW as an indicator of inflammation and oxidative stress in the body. Förhécz et al. (2009) observed that RDW correlated with various neuroinflammatory markers, including interleukin-6, C-reactive protein, and soluble tumor necrosis factor receptor I. The possible explanation is that elevated inflammatory factors may shorten the half-life of red blood cells, disrupt their membrane deformability, and impair their maturation, leading to the release of immature red blood cells into peripheral circulation (Lippi et al., 2009; Bazick et al., 2011). RDW is closely related to the lifespan of red blood cells. Prior research indicates that HbA1c or the ratio of HbA1c to glycated albumin might serve as indicators for assessing mean erythrocyte age (Koga et al., 2023). Therefore, we collected Mrbc calculated based on HbA1c to assess the effect of red blood cell mean lifespan on cognition. Since diabetes significantly impacts HbA1c levels, the Mrbc formula is more applicable to non-diabetic patients. Nevertheless, no significant differences in Mrbc were observed between the PSCI and PSNCI groups, even within the non-diabetic population (see Supplementary Figure 1). Possible reasons include that there are many influencing factors of PSCI and the influence of indicators related to red blood cell lifespan is limited. Additionally, stroke patients often display various complications, and some individuals in the non-diabetic population exhibit insulin resistance, which can influence HbA1c levels. Finally, the small sample size may limit the interpretation of results. Additional analyses revealed a weak positive correlation between HRR and Mrbc in non-diabetic participants (r = 0.176, *P* = 0.013) (see Supplementary Figure 2A). However, no significant correlations were found between Mrbc and hemoglobin (r = 0.137, *P* = 0.055) or Mrbc and RDW (r = −0.111, *P* = 0.120) (see Supplementary Figures 2B, C). This suggests that HRR is more strongly associated with red blood cell lifespan than a single indicator. Future research should confirm the utility of HRR in evaluating the lifespan of red blood cells in non-stroke individuals. Serum selenium, an essential component of the antioxidant defense system, exhibits a negative correlation with RDW (Semba et al., 2010). Elevated RDW levels may indicate severe oxidative stress and are potentially linked to neuronal damage in cerebral ischemia and reperfusion injury. In addition, inflammation and oxidative stress jointly contribute to the progression of atherosclerosis (Marchio et al., 2019), representing a crucial pathological mechanism underlying cognitive decline (Liu et al., 2017). These interpretations can be applied to the observed relationship between HRR and PSCI.

There are several limitations to this study. First, we only collected HRR data at admission and lacked relevant data during follow-up, so we were unable to analyze the impact of dynamic changes in HRR on PSCI. Second, study participants had low baseline NIHSS score, suggesting that the findings may only apply to patients with mild to moderate stroke. Third, the exclusion of patients with severe aphasia or dysarthria may introduce selection bias. Fourth, the lack of laboratory data on iron levels and other hematopoietic raw materials made it impossible to analyze the effect of specific anemia types and hematopoietic raw materials on the results. Future studies should incorporate these indicators into analyses to better guide clinical complementary therapy. Finally, we only assessed cognitive function through MMSE and future studies need more detailed neuropsychological assessments to assess cognitive function.

## 5 Conclusion

In summary, our findings suggest that low baseline HRR is associated with cognitive impairment one month after stroke. Compared to other markers, HRR is cost-effective and easy to access. Early clinical attention to this metric can effectively identify patients at high risk for PSCI.

## Data Availability

The raw data supporting the conclusions of this article will be made available by the authors, without undue reservation.
