# Supplementary material for: Relationship between the hemoglobin-to-red cell distribution width ratio and post-stroke cognitive impairment: a prospective study
Source: Front Aging Neurosci. 2025 Apr 30;17:1552956. doi: 10.3389/fnagi.2025.1552956 (PMC12075231; doi:10.3389/fnagi.2025.1552956)
Supplement: Supplementary file 1 [file Table_1.docx]

**Supplementary Figure 1 Comparison of mean erythrocyte age between PSCI group and PSNCI group in non-diabetic participants**

There was no significant difference in Mrbc between the PSCI and PSNCI groups in non-diabetic participants [54.63 (47.50-60.97) days vs. 54.63 (49.88-62.16) days, *P* = 0.639] . (Supplementary Figure 1)


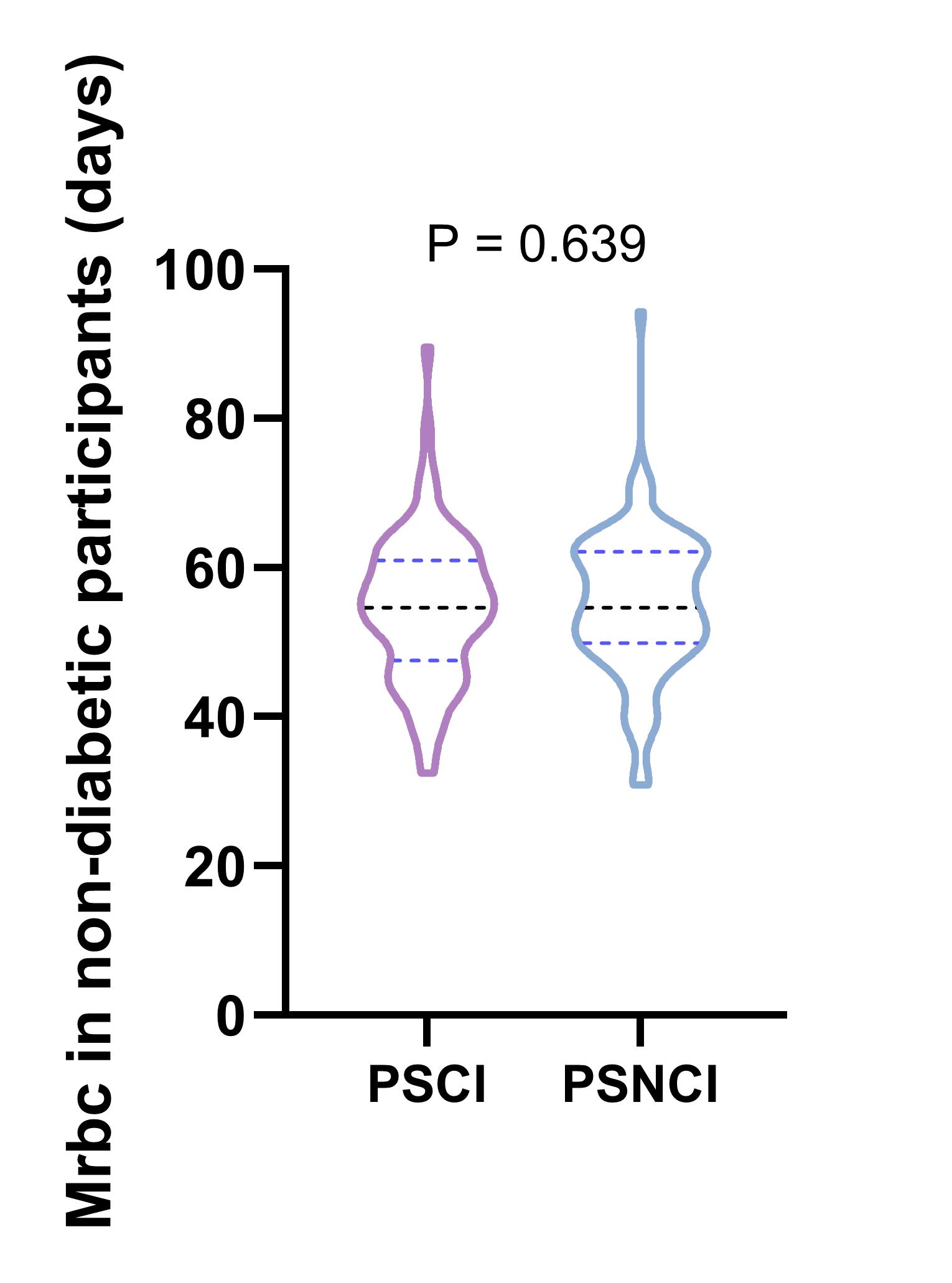


Supplementary Figure 1: Comparison of mean erythrocyte age between PSCI group and PSNCI group in non-diabetic participants. Mrbc, mean erythrocyte age; PSCI, Post-stroke cognitive impairment; PSNCI, Post-stroke non-cognitive impairment.

**Supplementary Figure 2：Correlation analysis between Mrbc and HRR, hemoglobin, RDW**

There was a positive correlation between Mrbc and HRR in non-diabetic participants (r = 0.176, *P* = 0.013). However, no significant correlations were found between Mrbc and hemoglobin (r = 0.137, *P* = 0.055) or Mrbc and RDW (r = -0.111, *P* = 0.120). (Supplementary Figure2)


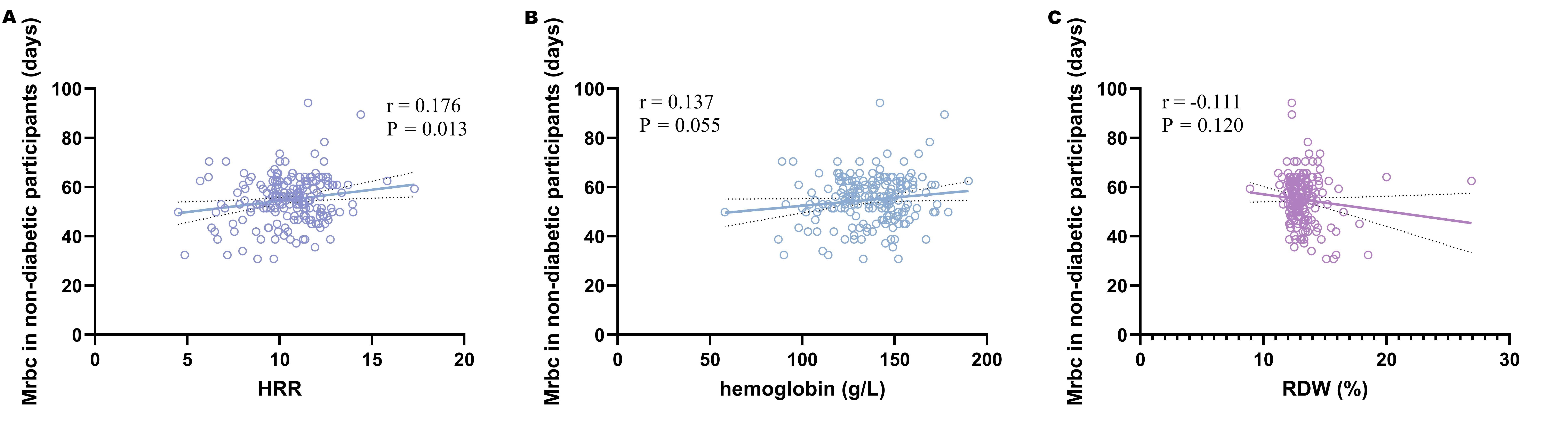


Supplementary Figure 2: Correlation analysis between Mrbc and HRR, hemoglobin, RDW. A: Correlation analysis of Mrbc and HRR; B: Correlation analysis of Mrbc and hemoglobin; C: Correlation analysis of Mrbc and RDW; Mrbc, mean erythrocyte age; HRR, Hemoglobin-to-red cell distribution width ratio; RDW, Red cell distribution width.
